# A comparative study on photosynthetic characteristics and flavonoid metabolism between *Camellia petelotii* (Merr.) Sealy and *Camellia impressinervis* Chang &Liang

**DOI:** 10.3389/fpls.2022.1071458

**Published:** 2022-11-24

**Authors:** Xin Huang, Bo Qin, Lei Qin, Zhihong Peng, Shitou Xia, Yi Su, Kaidao Sun, Keqin Peng

**Affiliations:** ^1^ Hunan Provincial Key Laboratory of Phytohormones and Growth Development, Hunan Agricultural University, Changsha, China; ^2^ Forestry Research Institute of Guangxi Zhuang Autonomous Region, Nanning, China

**Keywords:** *C. impressinervis*, *C. petelotii*, flavonoid, light-stress, photosynthesis, transcriptomic analysis

## Abstract

*Camellia petelotii* (Merr.) Sealy and *Camellia impressinervis* Chang & Liang belong to the golden subgroup of *Camellia* (Theaceae). This subgroup contains the yellow-flowering species of the genus, which have high medicinal and ornamental value and a narrow geographical distribution. These species differ in their tolerance to high light intensity. This study aimed to explore the differences in their light-stress responses and light damage repair processes, and the effect of these networks on secondary metabolite synthesis. Two-year-old plants of both species grown at 300 µmol·m^-2^·s^-1^ photosynthetically active radiation (PAR) were shifted to 700 µmol·m^-2^·s^-1^ PAR for 5 days shifting back to 300 µmol·m^-2^·s^-1^ PAR for recovery for 5 days. Leaf samples were collected at the start of the experiment and 2 days after each shift. Data analysis included measuring photosynthetic indicators, differential transcriptome expression, and quantifying plant hormones, pigments, and flavonoids. *Camellia impressinervis* showed a weak ability to recover from photodamage that occurred at 700 µmol·m^-2^·s^-1^ compared with *C. petelotii.* Photodamage led to decreased photosynthesis, as shown by repressed transcript abundance for photosystem II genes *psbA, B, C, O,* and *Q*, photosystem I genes *psaB, D, E, H,* and *N*, electron transfer genes *petE* and *F*, and ATP synthesis genes *ATPF1A* and *ATPF1B*. High-light stress caused more severe damage to *C. impressinervis*, which showed a stronger response to reactive oxygen species than *C. petelotii.* In addition, high-light stress promoted the growth and development of high zeatin signalling and increased transcript abundance of adenylate dimethylallyl transferase (IPT) and histidine-containing phosphotransferase (AHP). The identification of transcriptional differences in the regulatory networks that respond to high-light stress and activate recovery of light damage in these two rare species adds to the resources available to conserve them and improve their value through molecular breeding.

## Introduction

*Camellia oleifera* which belongs to the same family as the golden subgroup of *Camellia* has been studied for photosynthesis because of its wide cultivation area and the development of related industries. The photosynthetic characteristics of *Camellia oleifera* have been measured, and it was found that the main functional leaf for photosynthesis in *Camellia oleifera* is the 2-year-old leaf, which has the highest chlorophyll content and photosynthetic rate (Liang et al, 1988; Li et al., 2010). In contrast, the golden subgroup of *Camellia* is a shade-tolerant plant and is susceptible to strong light stress. The physiological and ecological characteristics of the golden subgroup of *Camellia* are closely related to their extremely narrow geographical distribution (Wei et al., 2008; Yang et al., 2010), strong light is not conducive to the growth and development of the golden subgroup of *Camellia* and is an important factor limiting their population expansion (Wei et al., 2007), therefore, the in-depth study of different light intensities of the golden subgroup of *Camellia* can provide the theoretical basis for population migration and conservation by understanding the intrinsic metabolic changes and regulatory expression characteristics of the golden subgroup of *Camellia*.

In this paper, we chose *Camellia petelotii* and *C. impressinervis* as the experimental materials; since that *C. petelotii* distribution latitude is wide; *C. impressinervis* is more distantly related to others the golden subgroup of *Camellia*, and the distribution data show that the distribution area is narrower (Liu, 2015).

*Camellia petelotii* and *C. impressinervis* are members of the subgroup of tea plants that produce golden yellow flowers and possess valuable medicinal resources, especially in China (Song et al., 2011; Zhang et al., 2020). *Camellia petelotii* is an evergreen shrub that is distributed in a narrow region of South China and North Vietnam. *C. impressinervis* is an extremely rare germplasm resource that is produced through hybridization (Tang et al., 2006; Chai et al., 2018) and is only distributed in Longzhou County and Daxin County, Guangxi, China (22.3° - 22.8° N, 106° - 109° E). It grows at 130 m to 480 m above sea level, in a region with an annual average temperature of 21°C to 22.8°C, annual rainfall of 1260 mm to 1360 mm, and in soils of pH 5.5 to 7.5.

Based on the results of Chai et al. wild *C. petelotii* mainly grows in valleys or on shady slopes, and is poorly adapted to strong light. *Camellia impressinervis* has high ornamental value for its leaves and flowers (Liao et al., 2015). It prefers similar habitats to *C. petelotii* and grows well in shady and humid environments (Chai et al., 2018). However, during 5 days of monitoring of plants at 300 µmol·m^-2^·s^-1^ photosynthetically active radiation (PAR) (24°C, 12 h light, 12 h dark), it was found that the net photosynthetic rate of *C. petelotii* was significantly higher than that of *C. impressinervis*. Li F. et al. 2013, also found that the stomatal density of *C. impressinervis* (204 mm^-2^) was 1.7-fold higher than that of *C. pete-lotii* (120 mm^-2^).

Our previous studies showed that the concentrations of total flavonoids, saponins, and tea polyphenols were higher in the leaves of *C. impressinervis* than those of *C. petelotii* (Wang et al., 2018). Song et al. determined the polyphenol concentration and antioxidant capacity of *C. petelotii*, *C. impressinervis*, *C. tunghinensis*, and three other camellia germplasms. They found that the concentrations of peroxyl radical scavenging activity (ORAC), total phenolics (TP), proanthocyanidin (PA), hydrolyzable tannin, and condensed tannin were significantly higher in *C. impressinervis* than the five other species. Remarkably, the flavone concentration was about 10-fold higher in *C. impressinervis* than in *C. petelotii*. Flavonoids such as flavonols, dihydro flavonoids, chalcones, and anthocyanins are the plant secondary metabolites that vary widely in concentration among plants. They have complex and diverse structures, mainly based on a C6-C3-C6 structure (Iwashina, 2000; Wang et al., 2013). At present, flavonoids are widely used as natural compounds with antioxidant properties that prevent cell damage. Because *C. petelotii* leaves are rich in anthocyanins and flavanols with antioxidant and antibacterial effects, their extracts are used in China to reduce blood cholesterol, blood pressure, and cancer (Yang et al., 2018; He et al., 2019; Zhang et al., 2020).

Taken together, differences in the photosynthetic rate at 300 µmol·m^-2^·s^-1^, stomatal density, and photoprotective capacity between *C. petelotii* and *C. impressinervis* suggest that both the species show significantly different responses to the light intensity that can impact their growth and development. This study, therefore, deciphers the differential modulation of gene networks of *C. petelotii* and *C. impressinervis* in response to light stress and photo-damage recovery. Exposure of two-year-old leaves to high light intensity and the recovery at normal intensity triggered significant changes in the photosynthetic indicators and phytochemicals. Moreover, the transcriptome analysis identified a number of DEGs with significantly differential expression patterns between the two species. The crucial information, thus obtained, can be effectively used to breed new cultivars with increased tolerance against light stress.

## Materials and methods

### Plant materials

The test seedlings were 2-year-old *Camellia petelotii (Merr.)* Sealy and *Camellia impressinervis* Chang & Liang. The seedlings were to grow 8-10 leaves during the experiment.

In previous trials, the photosynthetic rate of *C. petelotii* leaves under gradient light intensity were carefully tested, and three light intensities of 700, 300, and 100 µmol·m^-2^·s^-1^ were set in the plant culture room for 7 days, it was found that under strong light stress (700 µmol·m^-2^·s^-1^), the thylakoid morphology is abnormal in the leaves, the antioxidant system is damaged, 300 µmol·m^-2^·s^-1^ is a relatively appropriate growth light condition and that strong light stress (700 µmol·m^-2^·s^-1^) causes damage to the photosystem of *C. petelotii.* (Huang et al., 2022)

Therefore three stress-repair trial phases were set up in the CONVIRON CG72 walk-in plant growth chamber, the initial phase at a light intensity of 300 (µmol·m^-2^·s^-1^), the stress phase at a light intensity of 700 (µmol·m^-2^·s^-1^), and the recovery phase at a light intensity of 300 (µmol·m^-2^·s^-1^). The chamber temperature was set to 25°C, with a humidity of 70%. The plants were treated for 5 days. Measurements were taken and leaves were harvested after 5 h of light exposure on day 2 of each treatment. Some leaves were kept fresh while others were snap-frozen in liquid nitrogen, depending on the intended use.

**Figure 1:**
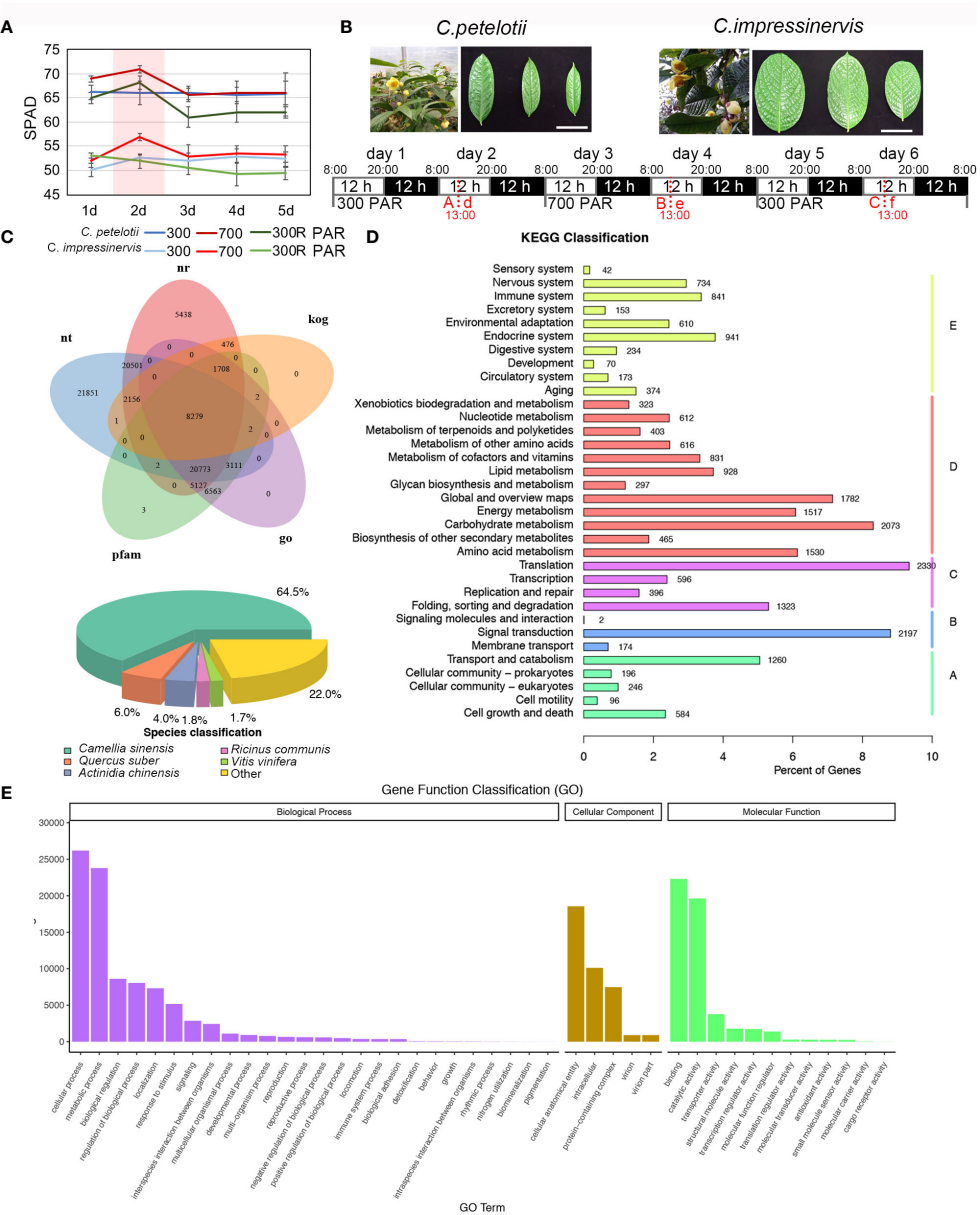
Gene functional annotation for a combined *Camellia petelotii* and *Camellia impressinervis* transcriptome. **(A)** Leaf SPAD values for *C. petelotii* and *C. impressinervis* exposed to 300 µmol·m^-2^·s-^1^ for 5 days, followed by 700 µmol·m^-2^·s^-1^ for 5 days and finally shifted back to 300 µmol·m^-2^·s^-1^ for 5 days. SPAD readings were taken each day. Bars show means ± s.d. (n = 3) **(B)** Leaves of *C. petelotii* and *C. impressinervis* are shown across the top of the panel. Scale bar = 50 mm. Based on the results in **(A)**, *C. petelotii* and *C. impressinervis* were exposed to 300 µmol·m^-2^·s^-1^ for 2 days, followed by 700 µmol·m^-2^·s^-1^ for 2 days and finally shifted back to 300 µmol·m^-2^·s^-1^ for 2 days. Photosynthetic measurements were taken and leave was harvested 5 h after the start of the light period (13:00) on day 2 of each treatment (red dotted lines). Leaf samples from *C. petelotii* were named A, B, and C, while leaf samples from *C. impressinervis* were named d, e, and f. Samples corresponding to 300 µmol·m^-2^·s^-1^ exposure (A and d), 700 µmol·m^-2^·s^-1^ exposure (B and e), and recovery at 300 µmol·m^-2^·s^-1^ (C and f) were used for transcriptome sequencing. **(C)** A Venn diagram of gene an-notations in the combined transcriptome from *C. petelotii* and *C. impressinervis*. Annotations were extracted from nt, NCBI non-redundant (nr), Eukaryotic Orthologous Groups (kog), Protein Families (Pfam), and Gene Ontology (GO) databases (top). Pie chart of the proportion of annotated genes in Camellia sinensis, Quercus suber, Actinidia chinensis, Ricinus communis, Vitis vinifera, and other plants (bottom). **(D, E)** KEGG and GO enrichment analysis, respectively, for annotated genes in the combined *C. petelotii* and *C. impressinervis* transcriptome.

### Determination of photosynthetic rate and photosynthetic pigment concentrations

Leaf photosynthetic rate was measured by gas exchange (LI-6400, LI-COR Inc., Lincoln, NE, United States), while F_o_ and Fm were measured using a pulse modulation chlorophyll fluorescence spectrometer (PAM 2500, LI-COR Inc., Lincoln, NE, United States). Pigments were extracted from fresh leaves in the dark at 24°C by grinding 0.3 g sample in 95% ethanol with quartz sand and calcium carbonate powder in a mortar and pestle until the tissue became white. After allowing it to stand for 5 min, the homogenate was filtered and diluted to 25 mL with 95% ethanol. The absorbance was determined at 665 nm, 649 nm, and 470 nm. The concentrations of Chl a, Chl b, and carotenoids were calculated according to the method of Wellburn and Lichtenthaler (1984).

### Transcriptome analysis

RNA extraction, sequencing, and bioinformatic analyses were done commercially from snap-frozen leaves (Novogene, Beijing, China). Messenger RNA was purified from total RNA using oligo-dT attached to magnetic beads. Fragmentation was carried out using divalent cations at elevated temperatures in a commercial buffer (Next First Strand Synthesis Reaction Buffer, New England Biolabs (NEB), Ipswich, MA, USA). First-strand cDNA was synthesized using random hexamer primers and M-MuLV Reverse Transcriptase (NEB). Second-strand cDNA synthesis was performed using DNA polymerase I and RNase H. Remaining overhangs were converted into blunt ends *via* exonuclease/polymerase activities. After adenylation of 3’ ends, an adaptor with a hairpin loop structure (Next Adaptor, NEB) was ligated onto the cDNA to prepare for hybridization. To select cDNA fragments of 250 bp to 300 bp in length, the library fragments were purified by magnetic bead separation (AMPure XP system, Beckman Coulter, Beverly, MA, USA). The size-selected, adaptor-ligated cDNA was subjected to uracil-specific excision (USER Enzyme, NEB) at 37°C for 15 min followed by 5 min at 95°C. Amplification by PCR was performed using a high-fidelity DNA polymerase, Universal PCR primers, and Index (X) Primer (Phusion, Thermo Fisher Scientific, Wal-tham, MA, USA). The PCR products were purified by magnetic bead separation (AM-Pure XP system, Beckman-Coulter) and library quality was assessed (Bioanalyzer 2100 system, Agilent, Santa Clara, CA, USA).

Clustering of index-coded samples was performed (cBot Cluster Generation System using the TruSeq PE Cluster Kit v3-cBot-HS, Illumina, San Diego, CA, USA) according to the manufacturer’s instructions. After cluster generation, libraries were sequenced (Hiseq TM4000, Illumina) to generate 150 b paired-end reads.

### Differential expression analysis

Differential expression analysis was performed (DESeq2 (Love et al., 2014) in R package 1.16.1). The resulting P-values were adjusted using the Benjamin and Hochberg approach for controlling the false discovery rate. Genes with an adjusted P-value < 0.05 were considered to be differentially expressed. The selection of differentially expressed genes was based on the FDR ≤ 0.001 and an absolute value of log2 relative expression level ≥ 1.

### Term enrichment analysis of differentially expressed genes

Case-specific analysis of differentially expressed genes (DEGs), including NCBI non-redundant (nr) (https://blast.ncbi.nlm.nih.gov/Blast.cgi), Eukaryotic Orthologous Groups (kog) (http://www.ncbi.nlm.nih.gov/COG/), Protein Families (Pfam) (http://pfam.xfam.org/), Gene Ontology (GO) (http://geneontology.org/) and KEGG pathway enrichment analysis, was done. The GO enrichment analysis was implemented through the cluster Profiler package in R. The GO terms that were differentially abundant with a corrected P-value ≤ 0.05 were considered to be significantly enriched in a set of DEGs. The KEGG database resource was used to assign high-level functions and utilities to the DEGs (http://www.genome.jp/kegg/).

### Determination of total flavonoid concentration

According to Chen et al. (2016), total flavonoid concentration (TFC) was determined spectrophotometrically.

A) sample pre-treatment

The samples to be tested were first killed at 105°C for 15 min, then dried at 60°C, crushed, and set aside.

B) sample extraction

We took the sample to be tested and ground it to a fine powder. We weighed about 0.3 g of ground sample and performed reflux extraction with 60% ethanol twice (20 mL each time), and filtered it. The filtrates were combined and evaporated to dryness. The dry filtrate was dissolved with 60% ethanol and fixed the volume to 25 mL.

C) standard curve drawing

We precisely measured 0 mL, 0.1 mL, 0.2 mL, 0.4 mL, 0.6 mL, 0.8 mL, 1.0 mL, 1.2 mL, and 1.6 mL of rutin reference solution in a 10 mL volumetric flask, and added 60% ethanol to make the final volume of 2.0 Ml. We added 0.5 mL 5% NaNO_2_ solution, and kept for 6 min. Then, 0.5 mL of 10% Al (NO_3_)_3_ solution was added, and kept for 6 min, and added 4 mL of 4% Al(NO_3_)_3_ and NaOH solution, with 60% ethanol to fill the volume to the marked line, and kept for 15 minutes. Finally, we determined the absorbance at 510 nm and drew the standard working curve.

D) sample determination

Accurately measured 0.5-2.0 mL of sample solution was added to a 10 mL volumetric flask. According to the method of drawing a standard working curve, after adding 0.5 mL of 5% NaNO_2_ solution, the absorbance was measured, and the rutin content was checked in the sample solution from the standard working curve, followed by calculation of total flavonoids in the sample.

### Determination of plant hormones

The samples were ground in liquid nitrogen to dry powder. The 1.5 g of powder was put in a glass test tube and added isopropanol-water-hydrochloric acid mixed extract into it, followed by shaking at low temperature for 30 min. Then, dichloromethane was added, followed by shaking at low temperature for 30 min and centrifugation at 13000 r/min for 5 min at low temperature. The lower organic phase was removed, protected from light, and dried with nitrogen. The organic phase was reconstituted with methanol (0.1% formic acid); centrifuged at 4°C for 10 min (13000 r/min), and the supernatant was taken to 0.22 µm filter membrane. For HPLC-MS/MS detection, the extraction process was performed on an ice box at 4°C throughout the operation. Methanol (0.1% formic acid) was used to prepare different gradients of IAA, ABA, Zeatin, and GA_3_ standard solutions for the solvent, and the standard curve in practice linear outliers was excluded from the equation. The data acquisition system mainly included high-performance liquid chromatography (High-Performance Liquid Chromatography, HPLC) (Agilent 1290, https://www.agilent.com/) and tandem mass spectrometry (Tandem mass spectrometry, MS/MS) (Applied Biosystems 6500 Quadrupole Trap, https://sciex.com.cn/). Mass spectral data were processed using the software Analyst.

### Determination of CAT, POD, and SOD activities

We took 0.5 g of leaves in different treatment groups, according to weight (g): volume (mL) ratio=1:5-10, and added 0.2 mol/L PBS buffer (PH 7.2-7.4). The homogenate was prepared under ice bath conditions, centrifuged at 2500 r/min for 10 min, and used the supernatant and diluted with PBS buffer. CAT enzyme activity was determined according to the method of Johansson and Borg (1988). POD activity was determined according to the Naz et al. (2021) method. The enzymatic activity of SOD was determined according to the method of He et al. (2007), comparing two procedures based on the autoxidation of pyrogallol.

### Plant RNA extraction and real-time RT-PCR

Total RNA was extracted using Trizol reagent (TAKARA, Japan) and used for reverse transcription (Transcriptor First Strand cDNA Synthesis Kit, TAKARA, Japan). Complementary DNA was diluted 10-fold for real-time RT-PCR. The PCR cycling was done under the following conditions: 95°C for 15 s, 56°C for 30 s, 72°C for 30 s, and 40 cycles (Applied Biosciences 7500 thermocycler, Thermofisher, Waltham, MA, USA). Primers used in this study are shown in **Supplemental Table S5**. the internal control gene is *Tub1*.

### Data analysis

Data were processed and plotted with Excel 2010 software, and statistical and ANOVA analyses were performed with SPSS 19.0 software.

## Results

### Differences in SPAD, photosynthetic pigment, and photoresponse parameters under the light stress

The responses of *C. petelotii* and *C. impressinervis* to changes in light intensity were examined in the leaves of 2-year-old plants. To determine the time course of the light response, plants were moved to a growth chamber at 300 µmol·m^-2^·s^-1^ light for 5 days, then transferred to 700 µmol·m^-2^·s^-1^ for 5 days, followed by recovery at 300 µmol·m^-2^·s^-1^ for 5 days. SPAD values were the highest on the second day of each light treatment. At each point, SPAD values were higher for *C. petelotii* than for *C. impressinervis* (**Figure 1A**). Considering that SPAD values remained stable from day 3 to day 5 for each light treatment, the physical and chemical indexes, and transcriptome changes were determined on the material harvested in the middle of the light period on day 2 after the start of each light treatment (**Figure 1B**). Plants were then moved to the next light treatment at the end of day 2 (**Figure 1B**). The concentrations of chlorophyll a and b (Chl a and Chl b) in *C. petelotii* leaves decreased during each of the three treatments of 300 - 700 - 300 R (µmol·m^-2^·s^-1^), while carotenoid (Cr) concentration only decreased during the 300 R µmol·m^-2^·s^-1^ treatment (**Supplemental Figure S1**). The highest concentrations of Chl a, Chl b, and Cr for *C. impressinervis* were observed during the 700 µmol·m^-2^·s^-1^ treatment, which decreased during plant recovery under 300 µmol·m^-2^·s^-1^. However, the reduction rate during recovery at 300 µmol·m^-2^·s^-1^ was smaller in *C. impressinervis* leaves than in *C. petelotii* leaves (**Supplemental Figure S1**). At the same time, the minimum fluorescence (Fo) in *C. petelotii* decreased at 700 µmol·m^-2^·s^-1^ and did not recover, while Fo in *C. impressinervis* was not affected by the changes in light intensity (**Supplemental Figure S2**). The maximum quantum yield of photosystem II (PSII), Fv/Fm, increased when *C. petelotii* was moved to higher light intensity and did not fully recover when moved back to the lower light (**Supplemental Figure S2**). In contrast, Fv/Fm did not change when *C. impressinervis* was moved to higher light but then decreased during recovery at 300 µmol·m^-2^·s^-1^.

### Sequence assembly and gene functional annotation for *C. petelotii* and *C. impressinervis* transcripts

Both *C. petelotii* and *C. impressinervis* lack a high-quality genome assembly to use as a reference. Therefore, transcriptome assemblies were constructed from RNA-seq reads obtained from leaves of *C. petelotii* (samples A, B, C, respectively, **Figure 1B**) and *C. impressinervis* (samples d, e, f, respectively, **Figure 1B**) exposed to three light treatments. A total of three assemblies were made (one for each species independently, and one combined transcriptome for both species) using Trinity to process all clean RNA-seq reads and assigned annotations (Grabherr et al., 2011). Based on the information available in NR, Swiss-Prot, KEGG, KOG, GO, NT, and Pfam databases, 131,060 unigenes (found in at least one database) were annotated. A total of 65% of the assembled transcripts were highly homologous to genes in *Camellia sinensis* (**Figure 1C**). In the KEGG classification system, the largest number of annotated transcripts were related to translation (2330), carbohydrate metabolism (2073), and signal transduction (2197) (**Figure 1D**). Moreover, the unigenes were most highly represented by GO terms in functions associated with cellular and metabolic processes in the biological processes category and with binding, catalytic activity, and transporter activity in the molecular functions category (**Figure 1E**).

### Interspecific differentially expressed genes

**Figure 2:**
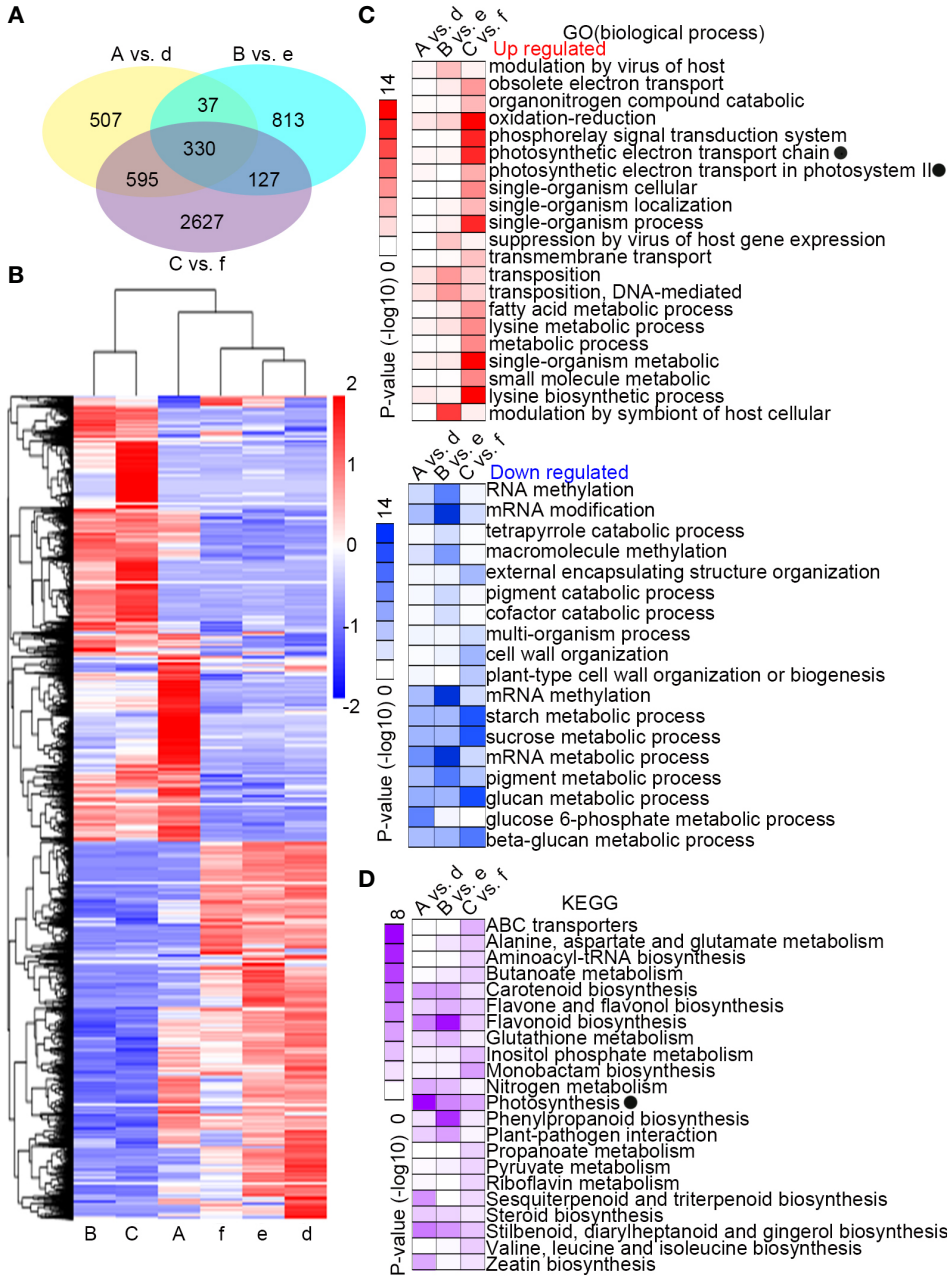
Differential response in gene expression to changing light intensity between *Camellia petelotii* and *Camellia impressinervis*. **(A)** Venn diagram of differentially expressed genes (DEGs) in a comparison between *C. petelotii* samples A, B, and C with *C. impressinervis* samples d, e, and f, respectively. See legend to **Figure 1B** for growth conditions and sample treatments. **(B)** Cluster analysis of the relative expression levels of the six groups in panel **(A)**. **(C)** GO enrichment analysis of DEGs from comparisons described in panel **(A)** Intensity of red and blue color indicates the statistical significance of the enrichment in DEGs that were up-regulated or down-regulated, respectively, in *C. petelotii* relative to *C. impressinervis*. **(D)** KEGG enrichment analysis of the DEGs from comparisons described in panel **(A)** Intensity of color indicates the statistical significance of the enrichment in *C. petelotii* relative to *C. impressinervis*. GO and KEGG terms associated with photosynthesis are marked (black dots).

Differentially expressed genes (DEGs) were identified with a log2 relative expression level ≥ 1 and ≤ −1 (p-value ≤ 0.05) in pairwise comparisons of samples A with d, B with e, and C with f. There were more unique DEGs between the two species during the recovery period at 300 µmol·m^-2^·s^-1^ (C vs. f, 2627 DEGs) than after the treatment at 300 µmol·m^-2^·s^-1^ (A vs. d, 507 DEGs) or at 700 µmol·m^-2^·s^-1^ (B vs. e, 813 DEGs) (**Figure 2A**). Cluster analysis of the DEGs by relative expression level showed similarity in expression trends within a species over the treatments, but not within treatments across species (**Figure 2B**). Gene Ontology enrichment analyses were performed with the up-regulated or down-regulated DEGs (**Figure 2C**). The enrichment was most significant between samples C and f. The functions that were most highly enriched in the up-regulated genes were involved in the photosynthetic electron transport chain, oxidation-reduction, and lysine biosynthetic process, while the most highly-enriched functions in the down-regulated genes were related to starch, sucrose, and glucan metabolism. The most significantly enriched functions among the down-regulated genes in B vs.e were related to methylation, metabolism, and modification of mRNA. KEGG enrichment analysis of the DEGs between species for each treatment showed significant enrichment signals in functions associated with photosynthesis and flavonoid biosynthesis (**Figure 2D**).

### Transcriptome differences in photosynthesis pathway genes

**Figure 3:**
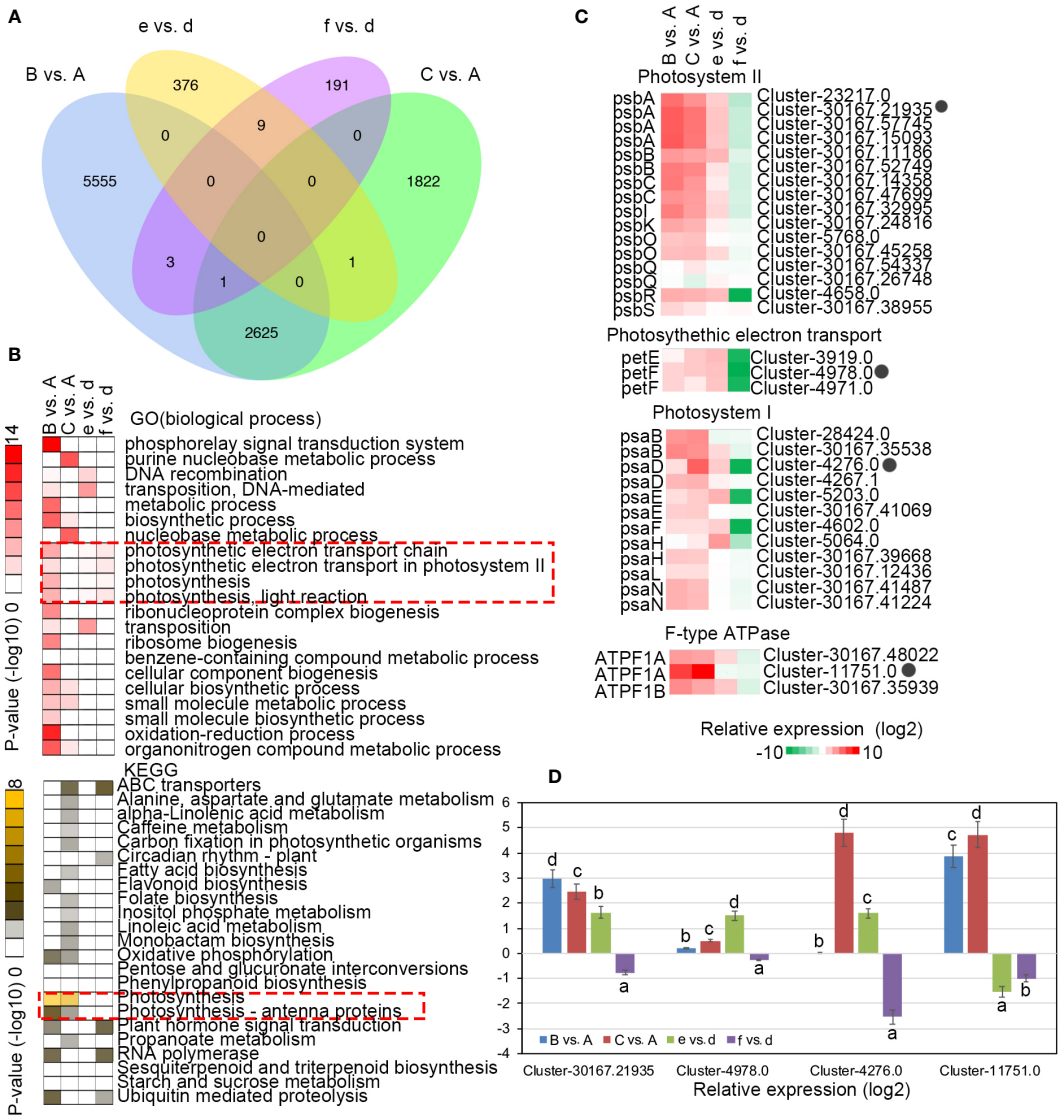
Differential expression of representative genes involved in photosynthesis in response to changing light intensity in *Camellia petelotii* and *Camellia impressinervis.* **(A)** Venn diagram of differentially expressed genes (DEGs) in response to changes in light intensity in *C. petelotii* samples B and C compared to sample A, and *C. impressinervis* samples e and f compared to sample d. See legend to **Figure 1B** for growth conditions and sample treatments. **(B)** GO and KEGG term enrichment analysis for DEGs identified in the comparisons described in panel **(A)** Color intensity indicates the statistical significance of the enrichment. **(C)** Relative expression of DEGs involved in photosynthesis is identified in the comparisons described in panel **(A)** Relative expression is shown on a gradient scale from strongly repressed (dark green) to strongly induced (dark red). **(D)** Relative expression profiles of four genes in the leaves of *C.petelotii* and *C. impressinervis* identified in the comparisons described in panel **(A)** were determined by real-time RT-PCR. All profiles were normalized to the expression level of tubulin in *C. petelotii* and *C. impressinervis*. All bars represent means ± s.d. (n = 3 biological replicates). Different letters above the bars indicate significant differences within a gene (P < 0.05) by Duncan’s test.

To further understand the differences in the transcriptional responses related to light stress response and recovery, DEGs within *C. petelotii* and *C. impressinervis* were identified by comparing sample A individually with samples B and C, and sample d individually with samples e and f, respectively (**Supplemental Tables S1**–**S4**). *Camellia petelotii* had more DEGs that respond to light stress (A vs. B, 8184 DEGs) and during the recovery process (A vs. C, 4449 DEGs) than *C. impressinervis* (d vs. e, 386 DEGs; d vs. f, 204 DEGs) (**Figure 3A**). GO and KEGG enrichment analysis showed that the overlap in response to changing light levels in the two species was largely restricted to the photosynthesis process (**Figure 3B**). The DEGs in these enriched groups included, the photosystem II reaction center chlorophyll-binding protein D1 (PsbA), subunit CP47 (PsbB), subunit CP43 (PsbC), an extrinsic subunit (PsbO), and the oxygen-evolving complex (PsbQ). Subunit D1 (PsaB), subunit II (PsaD), subunit VI (PsaH), and the only subunit of thylakoid lumen (PsaN) of the photosystem I reaction center. And photosynthetic electron transport protein (PetF) and subunits of the F1F0-ATPase synthase (ATPF1A; ATPF1B) (**Figure 3C**). Most of these genes were significantly up-regulated in the light stress response in both species. However, in *C. petelotii*, these genes remained significantly up-regulated in the recovery phase. In contrast, they remained comparatively low in *C. impressinervis*. Four genes that showed the most significant change in expression were selected to confirm the trends by real-time RT-qPCR analysis (**Figure 3C**). The results of RT-qPCR were highly consistent with the transcriptome data (**Figure 3D**). In general, *C. petelotii* and *C. impressinervis* have different transcriptional responses to light stress and photodamage recovery.

### Expression profiles for flavonoid biosynthesis pathways

**Figure 4:**
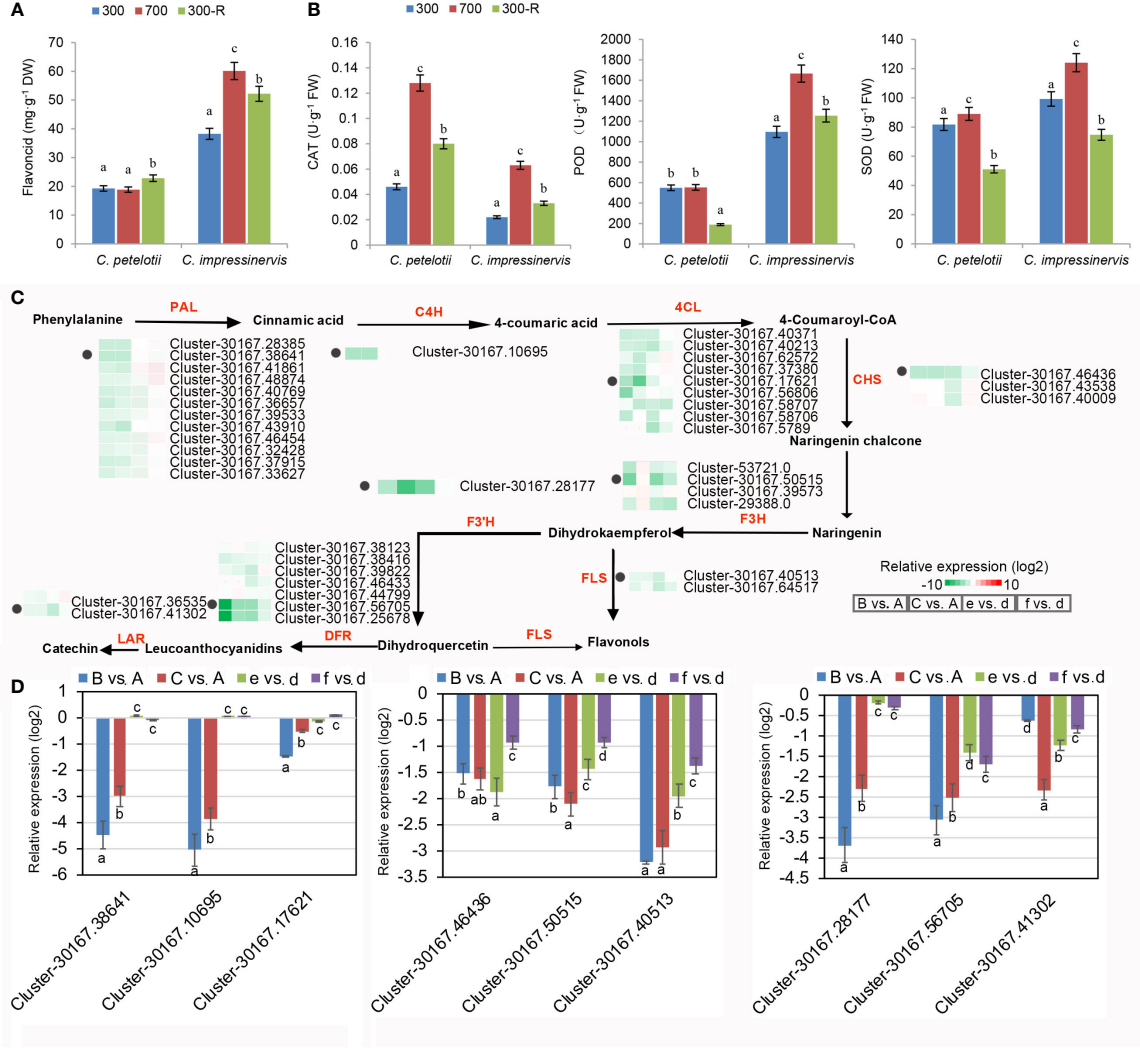
Differential expression of representative genes involved in flavonoid biosynthesis in response to changing light intensity in *Camellia petelotii* and *Camellia impressinervis*. **(A)** Total leaf flavonoid concentration in *C. petelotii* and *C. impressinervis* treated as described in the legend to **Figure 1B**. Data are shown as means ± SD (n=3), Different letters over bars indicate significant differences within a species by Duncan’s test (P < 0.05). **(B)** The concentration of leaf catalase (CAT), peroxidase (POD), and superoxide dismutase (SOD) activities. **(C)** Relative expression profiles of differentially expressed genes involved in flavonoid biosynthetic pathways in response to changes in light intensity in *C. petelotii* samples B and C compared to sample A, and *C. impressinervis* samples e and f compared to sample d. See legend to **Figure 1B** for growth conditions and sample treatments. Relative expression is shown on a gradient scale from strongly repressed (dark green) to strongly induced (dark red). Enzyme names are shown in red: phenylalanine ammonialyase (PAL), cinnamate-4-hydroxylase (C4H), 4-coumarate CoA ligase (4CL), chalcone synthase (CHS), flavanone-3-hydroxylase (F3H), flavanone-3’-hydroxylase (F3’H), dihydroflavonol 4-reductase (DFR), flavonol synthase (FLS) and leuanthocyanidin reduc-tase (LAR). Gene expression profiles confirmed by real-time RT-PCR are indicated (black dots). **(D)** Relative expression profiles of nine representative genes from panel C were determined in the leaves of *C. petelotii* and *C. impressinervis* by real-time RT-PCR. All profiles were normalized to the ex-pression level of tubulin in *C. petelotii* and *C. impressinervis*. In panels A, B and D, bars represent means ± s.d.(n = 3 biological replicates). Different letters above or below the bars indicate significant differences within a gene (P < 0.05) by Duncan’s test.

Leaves of *C. petelotii* and *C. impressinervis* are rich sources of flavonoids, which have a very prominent role as antioxidants. However, leaves of *C. petelotii* showed lower flavonoid contents at all treatments as compared to *C. impressinervis* (**Figure 4A**). The flavonoid concentration of *C. petelotii* leaves was unchanged after shifting to 700 µmol·m^-2^·s^-1^ and increased by about 15% after restoring the plants to 300 µmol·m^-2^·s^-1^. The flavonoid contents of *C. impressinervis* leaf were nearly doubled when plants were shifted to 700 µmol·m^-2^·s^-1^ and then declined during recovery at 300 µmol·m^-2^·s^-1^ (**Figure 4A**). The activities of the antioxidant enzymes, CAT, POD, and SOD were increased during high-light stress at 700 µmol·m^-2^·s^-1^ and decreased during the recovery at 300 µmol·m^-2^·s^-1^(**Figure 4B**). The activities of the antioxidant enzymes POD and SOD were significantly lower in leaves of *C. petelotii* than those of *C. impressinervis* (P < 0.05), but the CAT activity was significantly higher in leaves of *C. petelotii* than those of *C. impressinervis* (P < 0.05). The magnitude of these changes was accompanied by changes in the expression of a large number of genes related to flavonoid synthesis during a light stress response followed by damage repair and was consistent with the GO and KEGG enrichment analysis of the DEGs, showing significant enrichment in the oxidation-reduction process and flavonoid bio-synthesis during the light treatments (**Figures 2C, D**). The expression clustering of DEGs for nine gene families involved in flavonoid biosynthesis showed that most of these family members were repressed under light stress (**Figure 4C**). This down-regulation was more pronounced for *C. petelotii* than for *C. impressinervis*. The transcript abundance for the members of each of the gene families was confirmed by real-time RT-PCR (**Figure 4D**). Their expression levels were highly consistent with the RNA-seq data. Taken together, these results show that light stress affected flavonoid synthesis and antioxidant capacity in both species.

### Transcriptome differences in zeatin biosynthesis and signal transduction pathway

**Figure 5:**
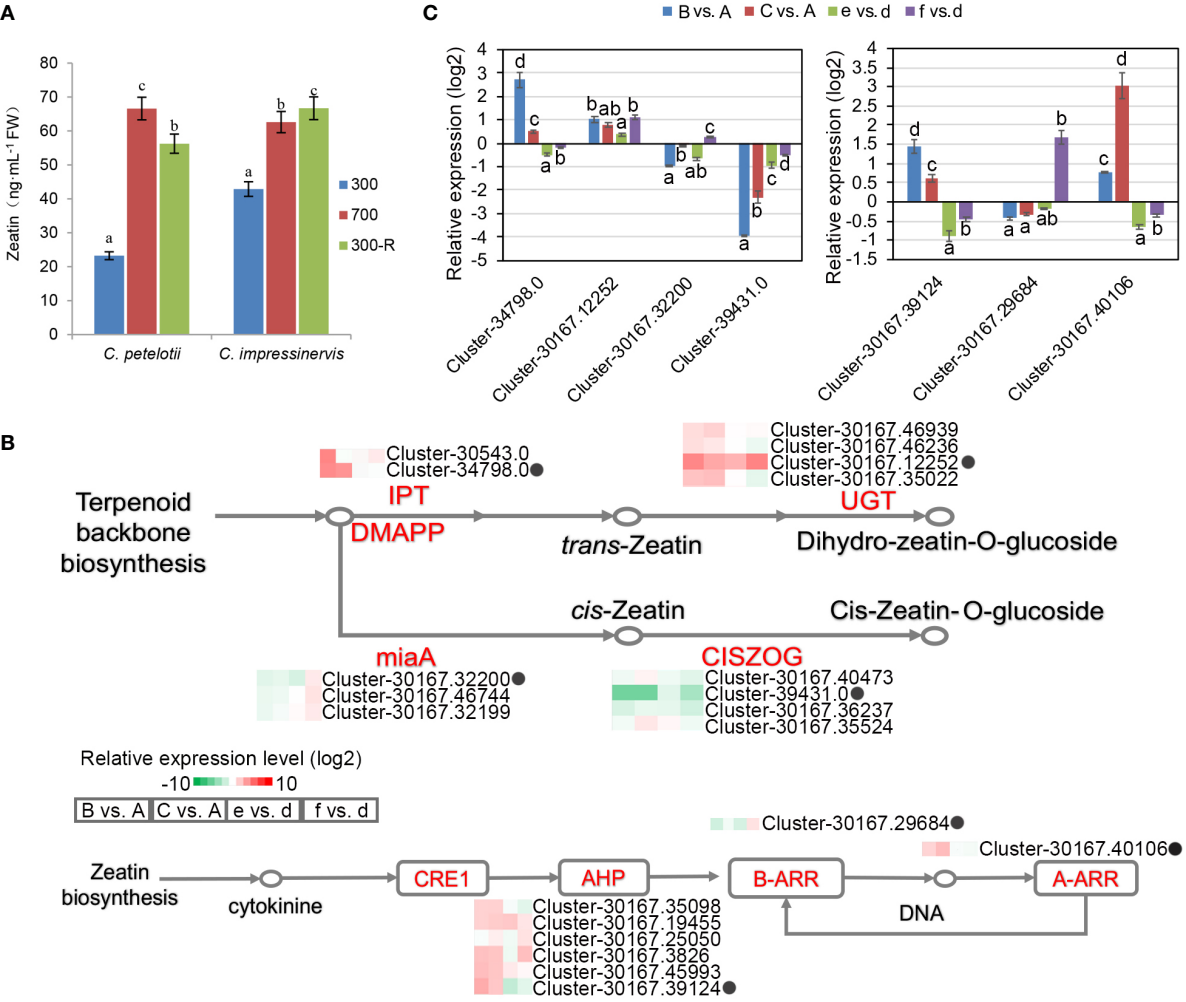
Differential expression of representative genes involved in zeatin biosynthesis and response in re-sponse to changing light intensity in *Camellia petelotii* and *Camellia impressinervis*. **(A)** Leaf zeatin concentrations in *C. petelotii* and *C. impressinervis* were treated as described in the legend in **Figure 1B**. Data are shown as means ± SD (n=3), Different letters over bars indicate significant differences within a species by Duncan’s test (P < 0.05). **(B)** Relative expression profiles of differentially expressed genes involved in zeatin biosynthesis and signal transduction in response to changes in light intensity in *C. petelotii* samples B and C compared to samples A, and *C. impressinervis* samples e and f compared to sample d. See legend to **Figure 1B** for growth conditions and sample treatments. Relative expression is shown on a gradient scale from highly repressed (dark green) to highly induced (dark red). Enzyme names are adenylate dimethylallyl transferase (IPT), UDP-glucosyltransferase (UGT), tRNA dimethylallyl transferase (miaA), cis-zeatin O-glucosyltransferase (CISZOG), histidine-containing phosphotransferase (AHP), and two-component response regulator family members (A-ARR, B-ARR), Gene expression profiles confirmed by real-time RT-PCR are indicated (black dots). **(C)** Relative expression profiles of seven representative genes from panel B were determined in the leaves of *C.petelotii* and *C. impressinervis* by real-time RT-PCR. All profiles were normalized to the expression level of tubulin in *C.petelotii* and *C. impressinervis*. Bars represent means ± s.d. (n = 3 biological replicates). Different letters above or below the bars indicate significant differences within a gene (P < 0.05) by Duncan’s test.

The concentrations of the plant hormones zeatin, indole acetic acid (IAA), abscisic acid (ABA), methyl salicylic acid (MeSA), methyl jasmonic acid (MeJA), and gibberellic acid (GA) were determined in leaves (**Figure 5A**, **Supplemental Figure S3**). Each hormone showed a species-dependent leaf concentration depending upon their response to the light treatment. The concentrations of IAA, MeSA, and MeJA were higher in the leaves of *C. impressinervis* than in the leaves of *C. petelotii* under all conditions. On the contrary, the concentration of ABA was higher in the leaves of *C. petelotii* than those of *C. impressinervis* under all conditions. The leaf concentration of GA_3_ was the same in both species at 300 µmol·m^-2^·s^-1^, but it was higher in *C. petelotii* during the high-light stress and recovery phase. The concentrations of Zeatin, IAA, MeSA, and GA_3_ were increased in both species during their shifting from normal to high-light stress, while ABA concentration decreased. The changes in hormone levels during the recovery phase were variable.

The enrichment of functions associated with zeatin biosynthesis and its role in signal transduction among the DEGs (**Figure 2D**) led to a more thorough examination (**Figure 5B**). The expression of adenylate dimethylallyl transferase (IPT) and UDP-glucosyltransferase (UGT), which regulate the biosynthesis of trans-zeatin, was increased by light stress, while tRNA dimethylallyl transferase (miaA) and cis-zeatin O-glucosyltransferase (CISZOG) were repressed. Six histidine-containing phosphotransferases (AHP) and one two-component response regulator family member (A-ARR) were up-regulated, while a B-ARR family member was repressed in the light stress response and recovery process in *C. petelotii*. Similar trends were absent in *C. impressinervis*. As before, RT-qPCR validation of the RNA-seq results was highly consistent (**Figure 5C**). Taken together, light stress affected the synthesis and signal transduction pathways of plant hormones, especially zeatin, in *C. petelotii*.

**Figure 6:**
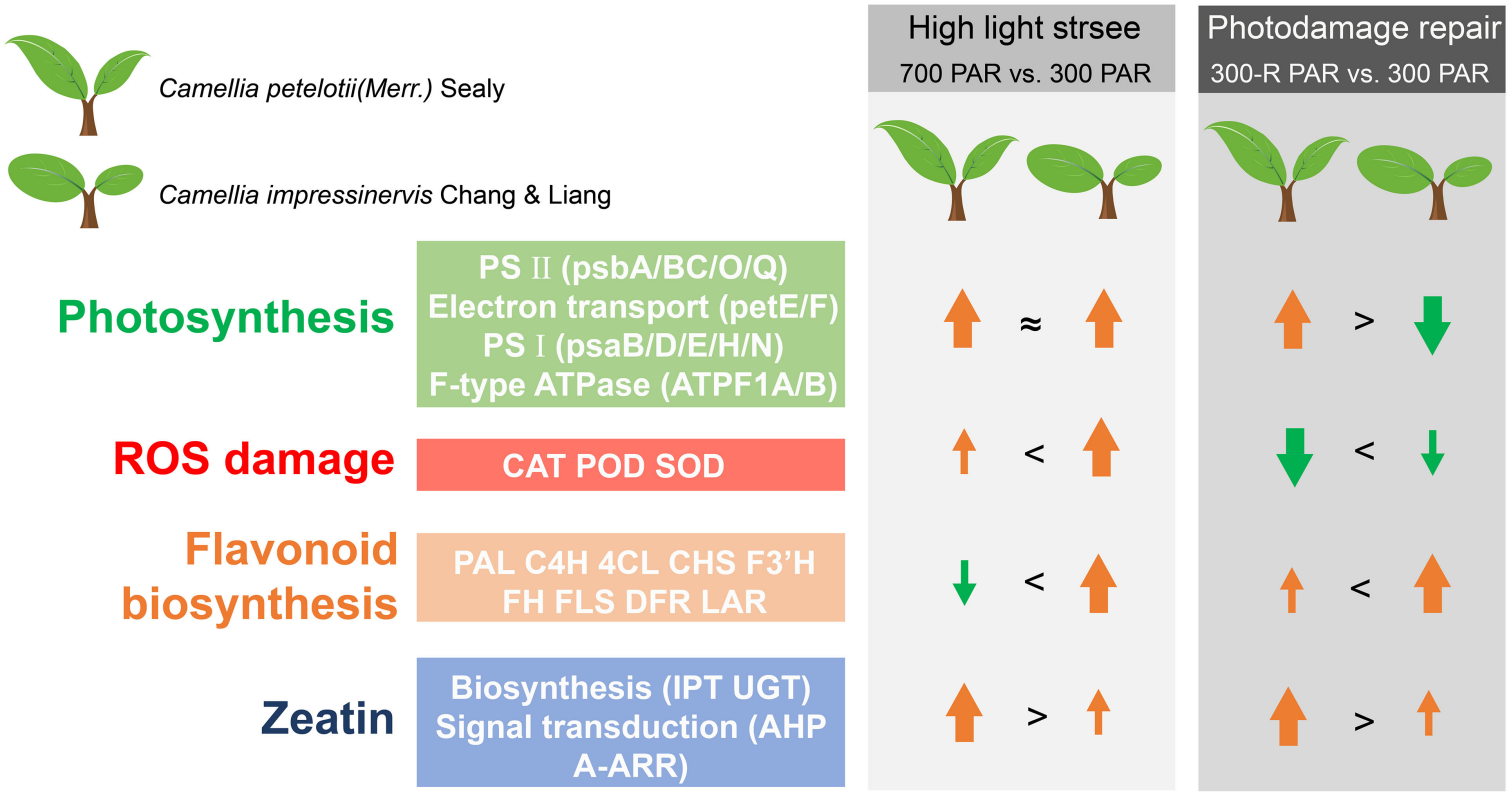
A model for the light response in leaves of *Camellia petelotii* and *Camellia impressinervis*.

## Discussion

### Material selection and accuracy of transcriptome data

The results demonstrated that the sampling used in this study was meaningful and the transcriptome data were accurate and representable. Moving 2-year-old *C. petelotii* and *C. impressinervis* plants from a growth cabinet with 12 hours of light exposure at 300 µmol·m^-2^·s^-1^ to high-light stress at 700 µmol·m^-2^·s^-1^ caused an increase in SPAD values at day 2, which declined by day 3. Thus, day 2 was optimal for leaf sampling to determine the differential response of both species to light stress.

When plants were subjected to intense light stress 700 µmol·m^-2^·s^-1^ and then restored to the optimum light intensity 300 µmol·m^-2^·s^-1^, the chlorophyll fluorescence intensity, phytochrome as well as physicochemical indicators such as hormone content and antioxidant enzyme activity of the leaves were measured and analyzed. Transcriptome measurements were carried out on the corresponding leaf tissues, aiming to further reveal the response mechanism of garlic leaves to light and the influence mechanism of strong light on the growth and development of garlic at the molecular level.

The assembled transcriptome was of suitable quality since 64.5% of the genes annotated in the unreferenced transcriptome were highly homologous to genes from *Camellia sinensis*. The gene expression and response profiles were distinct in the two species since there was a great similarity in the expression profiles within the three treatments of each species. The expression profiles determined by RNA-seq were accurate, as the real-time RT-qPCR analysis recapitulated and validated the transcript levels of 20 individual genes.

### Changes in photosynthetic gene expression patterns in response to light stress and recovery

*Camellia petelotii* and *C. impressinervis* possess very different regulatory mechanisms for modulating gene expression during light stress and stress-recovery periods. A large number of DEGs were obtained with significant expression differences between these two species due to light stress. According to GO and KEGG annotations, the gene set associated with photosynthesis was the most responsive both between the two species at the same treatment condition and within each species across the three treatments. The difference in response to the light treatments was also clear from the maximum quantum yield of photosystem II (Fv/Fm). *Camellia petelotii* was relatively unstressed by the changes in light regimens, with an Fv/Fm of about 0.8 in all treatments. This species seemed to be least stressed at 700 µmol·m^-2^·s^-1^, since it had a slightly higher Fv/Fm at this PAR intensity than at 300 µmol·m^-2^·s^-1^. However, *C. impressinervis* showed more stress responses at both 300 and 700 µmol·m^-2^·s^-1^, with an Fv/Fm value lower than 0.8, which was further dropped during the recovery phase. The differences in stress levels at low light intensity would likely cause differences in the long-term growth and development of these species.

The DEGs related to Photosystem II (*i.e.*, PsbA, B, C, O, Q), Photosynthetic electron transport (*i.e.*, petE, F), Photosystem I (*i.e.*, PsaB, D, E, F, H, L, N) and F-type ATPase (*i.e.*, ATPF1A, B) showed differential expression in *C. impressinervis* between 300 µmol·m^-2^·s^-1^ and recovery from 700 µmol·m^-2^·s^-1^ (**Figure 3C**). The RT-qPCR results of four of these genes showed their specific repression (**Figure 3D**). The transcript abundance for genes associated with PSII was consistent with the trends seen in PSII efficiency measured by Fv/Fm. In *C. petelotti*, the expressions of PSII-associated genes PsbA, B, C, O, and Q, as well as photosynthetic electron transport-associated genes petE and F, were higher in leaves of plants exposed to 700 µmol·m^-2^·s^-1^ and in the recovery phase than the plants exposed to the initial 300 µmol·m^-2^·s^-1^ condition. In *C. impressinervis*, the levels of these transcripts were lower in the recovery phase, associated with lower PSII efficiency (lower Fv/Fm), than the initial 300 µmol·m^-2^·s^-1^ treatment. The proteins encoded by these genes include PsbA (thylakoid located D1) and PsbB (chlorophyll A binding protein CP47), PsbC (CP43 protein), which are located in the PSII reaction center (Vaistij et al., 2000; Raszewski and Renger, 2008; Chotewutmontri and Barkan, 2018); PsbO, Q, and R, which are the extrinsic subunits in PSII oxygen-evolving complex (Allahverdiyeva et al., 2013); and PetE and F (cytochrome b559), which play important roles in the formation and stabilization of PSII structure by acting as electron carriers (Pospisil, 2011; Ido et al., 2012).

In *C. impressinervis*, the recovery phase repressed PSI transcripts, which encode the core protein PsaB, complex stability factor PsaD, subunit PsaE, calcium-dependent phosphorylation subunit PsaH and interactor between plastocyanin and the PSI complex psaN (Ihnatowicz et al., 2004; Liu et al., 2012; Stael et al., 2012). This repression was consistent with the decrease in the concentrations of Chl a and Chl b and carotenoids (**Supplemental Figure S1**). The increased transcript abundance for F1F0-ATPase subunits ATPF1A and B may allow an increase in photosynthetic electron transfer by reducing energy supply (St-Pierre et al., 2000; Bosetti et al., 2002). Taken together, during the switching of *C. petelotii* and *C. impressinervis* from 300 µmol·m^-2^·s^-1^ to 700 µmol·m^-2^·s^-1^, photosynthetic indexes and core regulatory genes were both up-regulated. However, during the recovery phase, the decrease in photosynthesis in *C. impressinervis* was consistent with the down-regulation of genes associated with photosynthesis.

From the results, it seems likely that there are no significant differences in photosynthetic indices between the two species in response to light stress, while the photodamage repair capacity of *C. impressinervis* was lower than that of *C. petelotii*. Higher light for a prolonged period (2 days) decreased the photosynthetic rate of *C. impressinervis*, probably by affecting the formation and stability of PSII and PSI complexes and altering electron transfer. The differences in the effects of light transitions on the photosynthetic apparatus are likely to be translated to long-term growth effects on plants, where *C. petelotii* would be favored in higher light environments than *C. impressinervis*.

### Response of flavonoid biosynthesis to light intensity

There are four orthologs in Arabidopsis encoding phenylalanine ammonia-lyase (PAL), the first enzyme in the flavonoid biosynthetic pathway. In contrast, over 31 PAL unigenes were identified in the *Camellia* spp. This finding suggests that an expansion in gene numbers explains why *Camellia* spp. is rich in flavonoids (Qin et al., 2020). Total leaf flavonoid concentration was significantly higher in *C. impressinervis* than that *C. petelotii*.

Flavonoids were significantly affected by the transitions in light exposure. Moving plants to 700 µmol·m^-2^·s^-1^ actually caused a reduction in the concentration of leaf flavonoids in *C. petelotii*, while a significant elevation was observed in *C. impressinervis*. During the recovery phase, the flavonoid concentration in *C. petelotii* increased within 2 days, and the flavonoid concentration in *C. impressinervis* decreased, but the concentration exceeded the level present before the higher light treatment in both species. A comparison of the transcript levels encoding the rate-limiting enzymes in the flavonoid biosynthesis pathway showed that the majority of genes were down-regulated under light stress, consistent with the decrease in flavonoid concentration during light stress repair. However, *C. impressinervis* showed similar levels of PAL transcripts during high-light stress and recovery phases. It is noteworthy that PAL activity is upstream of the rate-limiting enzymes, such as C4H, in the flavonoid synthesis pathway (Li X. et al., 2013). Therefore, flavonoid concentration was positively correlated with the activity of antioxidant enzymes, CAT, POD, and SOD, increasing with the shift of plants to 700 µmol·m^-2^·s^-1^. Thus, *C. impressinervis*, which was sensitive to higher light, appeared to respond to light stress and photodamage repair by stabilizing the rate of flavonoid synthesis and increasing the activity of antioxidant enzymes.

### Effects of light intensity on plant hormone biosynthesis and signal transduction

The trend of zeatin concentrations in leaves was distinct among the other hormones. Its concentration decreased significantly in *C. petelotii* during the photodamage repair phase, while it was continuously increased in *C. impressinervis*. From the transcript analysis, it appears that *C. petelotii* predominantly used terpenoids to synthesize *trans*-zeatin rather than *cis*-zeatin by increasing the expression of cytokinin synthase IPT. Overall, the transcript abundance for genes encoding elements of the zeatin signal transduction pathway was higher in *C. petelotii* than that in *C. impressinervis*. This included the up-regulation of *AHP* and *A-ARR* expression under high light, which could accelerate cell division and plant development. Both *AHP* and *A-ARR* are regulators of cytokinin signaling and their mutants in *Arabidopsis* display limited root elongation (Salome et al., 2006; Nishiyama et al., 2013). Moreover, mutants of *IPT* such as *atipt6-1* are small plants with light green leaves (Miyawaki et al., 2006). The cytokinin zeatin is an effective growth regulator in plant development and stress responses. Compared with *trans*-zeatin or other highly active cytokinins, *cis*-zeatin concentrations are typically lower and the expression level in plant tissues is limited (Gajdosova et al., 2011; Schafer et al., 2015). In summary, in contrast to photosensitive *C. impressinervis*, *C. petelotii* is likely to actively promote the synthesis and signal transduction of cytokinins, such as *trans*-zeatin, under higher-light stress to ensure normal plant growth and development.

## Conclusions

*Camellia impressinervis* is more sensitive to light intensity than *C. petelotii*, the ability to repair photodamage was relatively lower in *C. impressinervis* than in *C. petelotii*. This difference leads to decreased photosynthesis in *C. impressinervis*, likely through the decreased formation and stability of photosynthetic PSII and PSI complexes and the interconnecting electron transfer chain. It is suggested that the expression of the photosynthetic pathway in response to light stress and the repair process of light damage is differently regulated in *C. petelotii*. and *C. impressinervis*, and the expression of the photosynthetic pathway in *C. petelotii* of response to strong light stress was more significantly regulated in the prevention and repair of light damage. At the same time, *C. impressinervis* could increase its defense against reactive oxygen species arising from light stress by maintaining the rate of flavonoid biosynthesis and increasing the activities of antioxidant enzymes. It also appears that in response to increased light *C. petelotii* can actively promote the synthesis and signal transduction of cytokinins, represented here by trans-zeatin, to ensure normal plant growth and development (**Figure 6**).

## Data availability statement

The datasets presented in this study can be found in online repositories. The names of the repository/repositories and accession number can be found below: NCBI Sequence Read Archive database, PRJNA870239.

## Author contributions

Data curation, writing-original draft preparation, writing reviewing and editing, XH. Methodology, BQ. Software, LQ. Conceptualization, ZP. Reviewing, SX. Conceptualization, YS. Investigation, KS. Supervision, writing reviewing, and editing, KP. All authors contributed to the article and approved the submitted version.

## Funding

This study was supported by the Fundamental Research Funds for Guangxi Forestry Research Institute (201827).

## Acknowledgments

We thank professor Wuming Qin (Guangxi University) and all members (Hunan Provincial Key Laboratory of Phytohormones and Growth Development) for their helpful assistance during the research, and professor Patrick M. Finnegan (University of Western Australia) for helpful discussions.

## Conflict of interest

The authors declare that the research was conducted in the absence of any commercial or financial relationships that could be construed as a potential conflict of interest.

## Publisher’s note

All claims expressed in this article are solely those of the authors and do not necessarily represent those of their affiliated organizations, or those of the publisher, the editors and the reviewers. Any product that may be evaluated in this article, or claim that may be made by its manufacturer, is not guaranteed or endorsed by the publisher.

## Supplementary material

Supplement Figure S1 Leaf concentrations of chlorophyll a, chlorophyll b, and carotenoids in *Camellia petelotii* and *C. impressinervis* are treated as described in the legend to **Figure 1B** in the main text. Bars represent means± s.d (n = 3 biological replicates, 5 leaves for one biological replicate). Different letters over bars indicate significant differences within a species by Duncan’s test (P < 0.05).

Supplement Figure S2 Fo, Fv/Fm in leaves of *Camellia petelotii* and *Camellia impressinervis* treated as described in the legend to **Figure 1B** in the main text. Bars represent means ± s.d (n = 3 biological replicates). Different letters over bars indicate significant differences within a species by Duncan’s test (P < 0.05).

Supplement Figure S3 Concentrations of indole acetic acid (IAA), abscisic acid (ABA), methyl jasmonic acid (MeJA), methyl salicylic acid (MeSA), and gibberellic acid (GA3) in leaves of *Camellia petelotii* and *Camellia impressinervis* treated as described in the legend to **Figure 1B** in the main text. Bars represent means ± s.d (n = 3 biological replicates, 5 leaves for one biological replicate). Different letters over bars indicate significant differences within a species by Duncan’s test (P < 0.05).

Supplementary Table 1 Differentially expressed genes between sample B and sample A.

Supplementary Table 2 Differentially expressed genes between sample C and sample A.

Supplementary Table 3 Differentially expressed genes between sample e and sample d.

Supplementary Table 4 Differentially expressed genes between sample f and sample d.

Supplementary Table 5 Primers used for RT-PCR assays
