# Supplementary material for: A comparative study on photosynthetic characteristics and flavonoid metabolism between Camellia petelotii (Merr.) Sealy and Camellia impressinervis Chang &Liang
Source: Front Plant Sci. 2022 Nov 24;13:1071458. doi: 10.3389/fpls.2022.1071458 (PMC9762238; doi:10.3389/fpls.2022.1071458)
Supplement: Supplement Figure S1 [file Presentation_1.pdf]

Supplementary Material

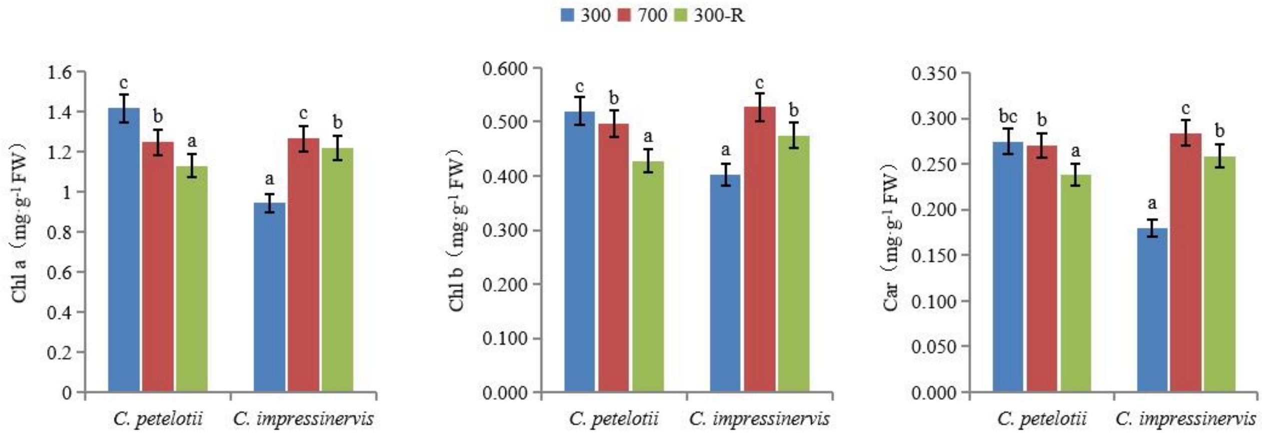

Supplementary Figure 1. Leaf concentrations of chlorophyll a, chlorophyll b and carotenoids in Camellia pete-lotiiand C. impressinervistreated as described in the legend to Figure 1B in the main text. Bars represent means± s.d (n = 3 biological replicates, 5 leaves for one biological replicate). Different letters over bars indicate significant differences within a species by Duncan's test (P < 0.05).

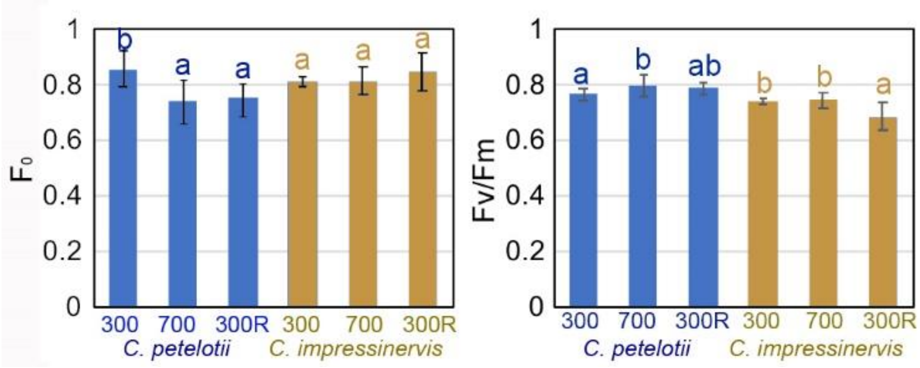

Supplementary Figure 2. F0, Fv/Fm in leaves of *Camellia petelotii* and *Camellia impressinervis* treated as described in the legend to Figure 1B in the main text. Bars represent means ± s.d (n = 3

biological replicates). Different letters over bars indicate significant differences within a species by Duncan’s test (P < 0.05).

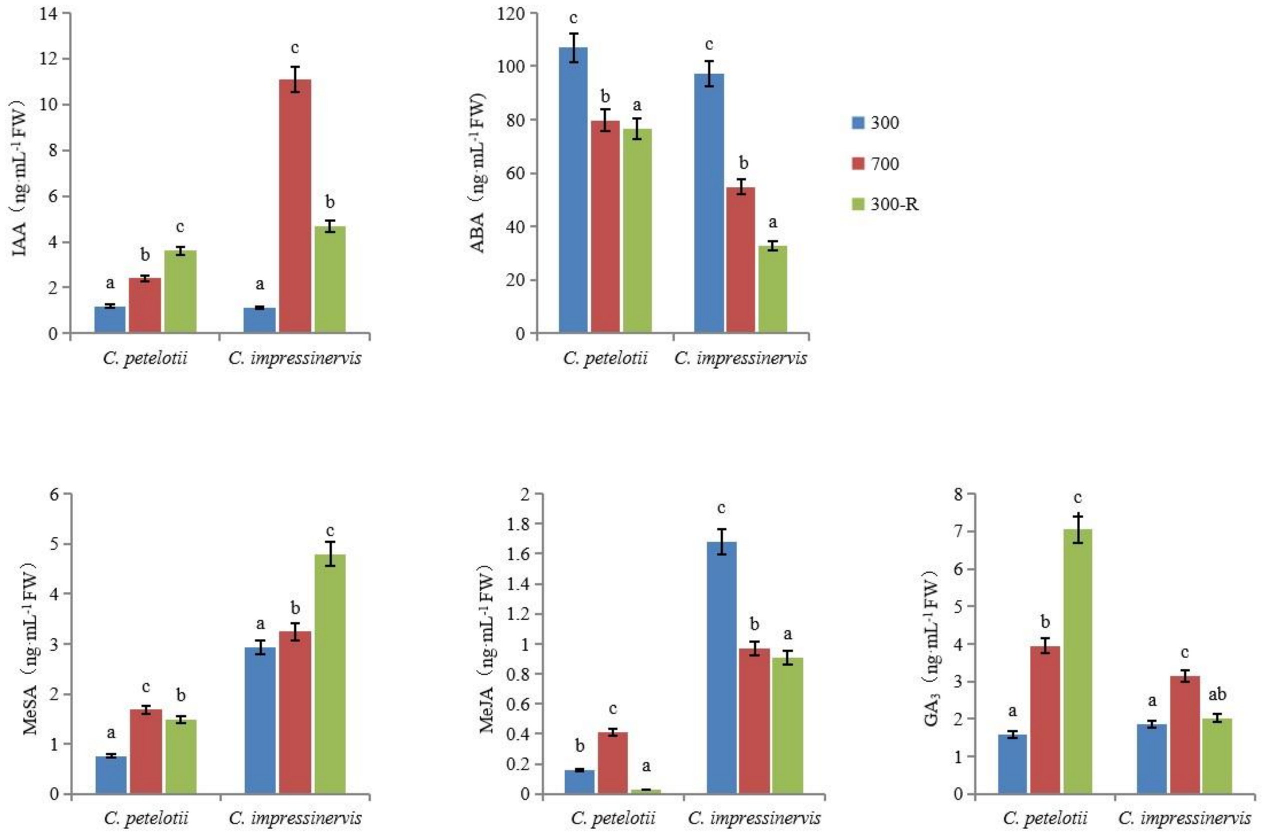

Supplementary Figure 3. Concentrations of indole acetic acid (IAA), abscisic acid (ABA), methyl jasmonic acid (MeJA), methyl salicylic acid (MeSA) and gibberellic acid (GA3) in leaves of *Camellia petelotii* and *Camellia impressinervis* treated as described in the legend to Figure 1B in the main text. Bars represent means ± s.d (n = 3 biological replicates, 5 leaves for one biological replicate). Different letters over bars indicate significant differences within a species by Duncan’s test (P < 0.05).
